# Molecular analysis of human Papillomavirus detected among women positive for cervical lesions by visual inspection with acetic acid/Lugol’s iodine (VIA/VILI) in Libreville, Gabon

**DOI:** 10.1186/s13027-016-0098-1

**Published:** 2016-09-07

**Authors:** Pamela Boundzanga Moussavou, Ismaël Hervé Koumakpayi, Andriniaina Andy Nkili-Meyong, Ingrid Labouba, Ulrich Bisvigou, Junie K. Chansi, Corinne Engohan-Aloghe, Frederic Dissanami, Nathalie Ambounda, Anne-Sophie Delannoy-Vieillard, Laure Diancourt, Dieudonne Nkoghe, Eric M. Leroy, Ernest Belembaogo, Nicolas Berthet

**Affiliations:** 1 Department of Zoonosis and Emerging Diseases, Centre International de Recherches Médicales de Franceville (CIRMF), Franceville, BP 769 Gabon; 2 Institut de Cancérologie de Libreville (ICL), Libreville, Gabon; 3 Centre Hospitalier Universitaire de Libreville (CHUL), Service de gynécologie obstétrique, Libreville, Gabon; 4 Institut de Recherches et de Développement (IRD), Maladies Infectieuses et vecteurs: Ecologie, génétique, Evolution et Contrôle (IRD 224 – CNRS 5290 6 UM1- UM2), Montpellier, France; 5 Institut Pasteur, Unité Environnement et risques infectieux, Cellule d’Intervention Biologique d’Urgence, Paris, France; 6 Ministère de la Santé, Direction général de la Santé, Libreville, Gabon; 7 Centre National de la Recherche Scientifique, UMR3569, Paris, France

**Keywords:** Human papillomavirus, Detection rate, Visual inspection, Molecular variant, Gabon

## Abstract

**Background:**

The human papillomavirus (HPV) is the causative agent of cervical cancer, which is the leading cancer-related cause of death for women in Sub-Saharan Africa. In 2013, the Gabonese Ministry of Health and the Sylvia Bongo Ondimba Foundation implemented cervical cancer screening programs based on the detection of cancerous lesions by visual inspection with acetic acid and/or Lugol’s iodine (VIA/VILI). This pilot study was set up to determine the HPV profile and analyze the nucleotide sequence variation of HPV16 circulating in patients with cervical abnormalities detected by VIA/VILI testing.

**Methods:**

The cervical abnormalities observed upon VIA/VILI were confirmed by liquid-based cytology for all tested women. Nested PCR using the MY09/11 and GP5+/6+ primer sets was used to detect HPVs present in the extracted DNA. HPV genotypes were determined after sequencing of amplicons based on a high-throughput sequencing approach. For isolates of the HPV16 genotype, the E6 gene and the long control region (LCR) were directly sequenced using Sanger method.

**Results:**

The study included 87 women who showed a positive VIA/VILI result. Cervical abnormalities were found in 40.23 % of women and 40 % were classified as high-grade lesions. The HPV detection rate was 82.9 % among women with abnormal cytology. Among all the identified high-risk HPV genotypes, HPV16, 18 and 33 were the most frequent. Multiple HPV infections were observed in 42.31 % of HPV-infected women. Analysis of the HPV16 sequence variation in the E6 gene and in the LCR showed that 85.3 and 14.7 % belonged to the African and European lineages, respectively. Among the African branch variants, Af2 was the most frequently identified in this study.

**Conclusion:**

This study offers the first report of the HPV detection rate and molecular epidemiology among Gabonese women with a positive result in a VIA/VILI screening test. Moreover, data on the HPV16 sequence variation confirm the predominance of African variants in high-grade lesions.

**Electronic supplementary material:**

The online version of this article (doi:10.1186/s13027-016-0098-1) contains supplementary material, which is available to authorized users.

## Background

In 2012, cervical cancer was the fourth most common cancer in women worldwide, with 528,000 new diagnosed cases, of which 70 % were recorded in developing countries [1]. Despite a global decrease, the incidence of recorded cervical cancer in Africa increased from 80,419 cases in 2008 to 99,038 cases in 2012. However, these statistics may be largely underestimated because only 8 % of the population in Sub-Saharan Africa is covered by cancer registries [2]. Moreover, in Sub-Saharan Africa, about one out of every two women who are diagnosed with cervical cancer will die of this disease [1, 3].

Cytology-based screening has considerably reduced the incidence of cervical cancer in countries that have implemented national programs. In Sub-Saharan Africa, these programs are difficult to organize due to limited infrastructures and funding, lack of trained staff, and a very strong competition with other national disease control programs, such as HIV, tuberculosis or malaria, and the fight against infant mortality. To overcome these issues and ensure early detection, cervical cancer screening programs based on visual inspection with acetic acid and Lugol’s iodine (VIA/VILI) can provide an immediate diagnostic evaluation [4]. Although the specificity of VIA/VILI seems to be lower in comparison with the Pap smear test, both tests have similar sensitivity [5]. VIA/VILI has the potential to become a very useful screening tool in developing countries even if it may be not adequate for primary screening [6].

The human papillomavirus (HPV) genotypes most frequently indicated as the causal factor of cervical cancer are HPV16, 18, 31, 33, 35, 45, 52 and 58 [7]. In Sub-Saharan Africa, the distribution of HPV genotypes in women remains under-documented. However, recent studies have shown that HPV16, 18 and 45 and HPV16 and 35 are the most frequent genotypes in West Africa and in South Africa, respectively [8, 9]. In Central Africa, the distribution of HPV genotypes among cervical cancer patients is available for some countries. In Gabon, the reported age-standardized incidence and mortality rates of cervical cancer were 19.9 and 8.4 per 100,000 women in 2014 [10]. A previous study of women either attending an antenatal clinic or presenting general symptoms related to genital diseases at a hospital revealed a high prevalence of HPV (46 %) in Libreville. The most frequent HPV genotypes then described were HPV16, 53 and 58 with 10, 12 and 11 % of prevalence, respectively [11]. In another study, the analysis of 39 HPV16 variants from patients with pre-cancerous lesions and invasive or micro-invasive carcinomas revealed the presence of only two African variants: Af1 (68 %) and Af2 (32 %) [12].

In January 2013, the Gabonese Ministry of Health, with the support of the Sylvia Bongo Ondimba Foundation, implemented cervical cancer screening programs at the *Institut de Cancérologie de Libreville* (ICL) and the *Centre Hospitalier Universitaire de Libreville* (CHUL). The cervical screening program is based on the detection of cancerous lesions and/or cervical abnormalities by VIA/VILI. Based on the World Health Organization (WHO) guidelines on “cervical cancer screening and treatment of precancerous lesions”, which recommends HPV detection, this pilot study was included in an epidemiologic surveillance program initially aimed to determine the detection rate and distribution of HPV genotypes among women with cervical abnormalities revealed by VIA/VILI testing [13]. We also analyzed the nucleotide variations in the E6 gene and the long control region (LCR) to identify the different HPV16 variants circulating in the Gabonese female population presenting cervical abnormalities.

## Methods

### Patient inclusion

This multi-center and cross-sectional study was carried out at two hospital centers, the ICL and the CHUL, located in Libreville (Gabon) which are screening reference centers for cervical and breast cancers. This screening program was open to women aged 25 and older. A total of 960 women residing in a suburb of Libreville participated in a screening program between January and March 2014. Only women whose VIA/VILI test results indicated cervical abnormalities during this period were included and signed specific informed consent forms that authorized the use of their cervical samples collected during a routine screening process. A general physical and detailed gynecological examination was carried out followed by VIA/VILI, screening with liquid-based cytology (LBC) and HPV testing. Exclusion criteria were pregnancy, severe gynecological bleeding and previous hysterectomy. The study was approved by the Medical Ethics Committee of Gabon (consent number PROT no. 0010/2013/SG/CNE), obtained the authorization of the Gabonese Ministry of Health (no. 00775/MS/CAB.M/SG/DGS) and of the Scientific Committee of the *Centre International de Recherches Médicales de Franceville* (CIRMF).

### Sample collection

The cervix was inspected after application of acetic acid and Lugol’s iodine according to the International Agency for Research on Cancer (IARC) criteria [14]. After a few minutes, exfoliated cells of the uterine ecto- and endocervix were collected by scraping with a cytobrush and then conserved in a Thin-Prep solution for LBC and HPV analyses. LBC samples were screened at Biomnis laboratory according to the Bethesda system 2001 and categorized as negative for intraepithelial lesions or malignancy (normal), atypical squamous cells of undetermined significance (ASC-US), low-grade squamous intraepithelial lesions (LSIL), high-grade squamous intraepithelial lesions (HSIL) and carcinoma.

### DNA extraction

A portion of Thin-Prep LBC samples were stored at -80 °C until the total genomic DNA extraction using the DNeasy Blood and Tissue kit (Qiagen, CA, USA) according to the manufacturer’s instructions. Extracted DNA was stored at -20 °C until subsequent analyses. DNA was quantified using Qubit® dsDNA BR Assay kit with the Qubit 2.0 fluorimeter (Life Technologies, CA, USA).

### L1 gene amplification for sequencing

The nested PCR assay was performed using two sets of degenerate primers (MY09/11 and GP5+/6+) (Additional file 1: Table S1). The first reaction was performed in 50 μl using 5 μl of template DNA, 1X PCR buffer (Invitrogen, CA, USA), 2.5 mM MgCl_2_, 0.125 μM of each deoxynucleotide triphosphate (dNTP), 0.25 μM of each consensus primer MY09/MY11 and 5 U of Taq DNA polymerase (Invitrogen). Thermal cycling performed with the following program: 10 min at 95 °C, 40 cycles of 45 s at 95 °C, 45 s at 55 °C and 40 s at 72 °C, with a final extension step at 72 °C for 8 min.

The second reaction was also performed in 50 μl including 5 μl of amplified DNA, 1X PCR buffer, 1.5 mM MgCl_2_, 0.05 μM each dNTP, 5 U Taq DNA polymerase and 0.4 μM each consensus primer GP5+/6+ tagged with an Illumina adapter sequence. Thermal cycling used the following program: 5 min at 94 °C, 40 cycles of 30 s at 94 °C, 30 s at 50 °C and 1 min at 72 °C, with a final extension step at 72 °C for 5 min. All primer sequences used are listed in Additional file 1: Table S1. The amplicon libraries and sequencing process are described in Supplementary Data.

### Generation of amplicon libraries

E-GP5+/6+ amplicons for sequencing were generated by PCR using modified primer sets, E-GP5+ and E-GP6+ (Additional file 1: Table S1). They were purified using magnetic beads (Agencourt® Ampure® XP) and indexed by PCR using the Nextera XT Index kit (Illumina, CA, USA) according to the manufacturer’s instructions. The resulting amplicon libraries were quantified using a Qubit fluorimeter with Qubit dsDNA HS Assay kit (Life Biotechnologies) and then with SYBR green quantitative PCR (Kapa Biosystems). Libraries were then normalized and multiplexed, resulting in a pool of libraries at a final concentration of 4 pM that was then mixed with a 4 pM PhiX control library. After a final heat-denaturation step (2 min at 96 °C), 600 ul of the pooled amplicon libraries were loaded into a MiSeq V2 Reagent v2 (300 cycles) cartridge (Illumina).

### Bioinformatics analyses for HPV genotyping

HPV genotypes were identified by running a python script on the reads. At the beginning, all paired-end reads were trimmed, filtered according to their quality and merged at the end. All merged reads were mapped against a local HPV database built from all viral sequences found in GenBank (NCBI-GenBank Flat File Release 191.0). To remove any risk of contamination among samples, all determined HPV genotypes represented by less than 2 % of the total number of reads in a sample were discarded. An HPV genotype was considered correctly identified if, among all reads related to the considered genotype, at least one read had a value of around 0 % of divergence. To validate a representative group of reads related to one specific HPV genotype, the distribution of the divergence percentage (DP) was determined based on the mean and the median of DPs of each HPV genotype as indicated in Additional file 1: Table S2.

### Sequence analysis of the E6 gene and LCR of HPV16

For HPV16-positive samples, the HPV E6 and LCR open reading frames were amplified by PCR as described in Yamada et al. [15]. Thermal cycling was based on the following program: 5 min at 95 °C, 40 cycles of 60 s at 95 °C, 60 s at 55 °C and 60 s (90 s for LCR) at 72 °C, with a final extension step at 72 °C for 5 min. The PCR mix contained 10 mM Tris (pH 8.3), 200 μM of each dNTP, 4 mM MgCl_2_, 0.125 μM of each sense-strand and anti-sense strand oligonucleotide primers, and 2.5 U of Taq in a final volume of 100 μl. All PCR products were sequenced using the BigDye Terminator v3.1 Cycle Sequencing kit (Applied Biosystems, ville?, pays?) according to the manufacturer’s protocol. The full length E6 and LCR ORFs were aligned with the reference HPV16 sequence (GenBank accession number: K02718) using multiple sequence alignment of the MEGA 5.2 package in the Clustal W program. The taxonomic nomenclature of molecular variants used in this study was adapted from Huertas-Salgado et al. [16].

### Statistical analysis

Statistical analyses were performed using IBM SPSS version 20.0.0. Summary statistics for age at study entry (n, mean, standard error, median, minimum and maximum) and cervical lesions (n, proportion) were produced. The associations between HPV infection and age groups and severity of cytological lesions were tested using the chi-squared test at the 95 % confidence level.

### Nucleotide sequence accession numbers

The E6 and LCR sequences are available in the DDBJ/EMBL/GenBank database under accession numbers KX296742 to KX296760.

## Results

### Participant characteristics

Among the 960 women participating in this screening program, only 93 showed positive VIA/VILI tests and were eligible. Six samples were excluded due to inconclusive cytological tests. Ultimately, 87 women were included in this study. The population was aged 24 to 80, the mean age of the cohort was 47.06 years (SD ± 12 years). LBC tests showed that 59.77 % (52/87) had normal cytology (Table 1). Among women with abnormal cytology, 20 % (7/35) had cervical cancer, 20 % (7/35) had HSIL, 22.9 % (8/35) had LSIL and 37.14 % (13/35) had ASC-US.

**Table 1:** Variable distribution according to HPV infection

	Total number of women	HPV-positive, n (%)	*P*-value
Age group			
[24–42]	34 (39.08)	8 (23.53)	< 0.05^a^
[43–61]	47 (54.02)	24 (51.06)	
[62–80]	6 (6.90)	3 (50)	
Cytology result			
Normal cytology	52 (59.77)	24 (46.15)	< 0.01^b^
Abnormal cytology	35 (40.23)	28 (82.9)	
ASC-US	13 (37.14)	7 (53.84)	
LSIL	8 (22.86)	7 (87.50)	
HSIL	7 (20)	7 (100)	
Carcinoma	7 (20)	7 (100)	

^a^Chi-squared = 6.47; df = 2

^b^Chi-squared = 36.00; df = 1

### HPV distribution among normal and dysplastic lesions

Nested PCR detected HPV DNA in 59.77 % (52/87) (CI: 49.5–70.1) of women. The mean age of women with positive HPV DNA was 48 (SD ± 12) years and 46.15 % (24/52) of women were aged 50–80. The association between HPV infection and abnormal cytology was significant (*p* ˂ 0.01) and increased with the severity of the cervical lesions (*p* < 0.01) (Table 1). Likewise, a significant difference was observed in the HPV infection rate among the three age groups (*p* ˂ 0.05) (Table 1).

The HPV infection profile was determined by high-throughput sequencing of GP5+/6+ amplicons. All detected HPV genotypes were classified into two categories: high-risk (HR) of cervical cancer (HPV16, 18, 31, 33, 35, 45 and 58) and low-risk (LR) of cervical cancer (HPV32, 62, 72, 81, 87 and 90). The detection rate of all HR-HPV was 94.64 % with a predominance of HPV16 (76.92 %), followed by HPV33 (2 5 %) and HPV18 (21.15 %). In total, 57.69 % (30/52) of cases that tested positive for HPV DNA contained a single HPV genotype (Table 2). HPV DNA was detected in 80 % of women with abnormal cytology. HPV16 was the most prevalent genotype reported in women with high-grade lesions: all HSIL (14) were infected by HR-HPV, among which 85.7 % HPV16 (12/14) and 14.3 % HPV18 (2/14) were found alone or in co-infection with other HPVs. HPV16 was detected alone in 42.86 % and 60 % of HSIL and carcinoma cases, respectively, whereas HPV18 was detected in a single infection in only one case of HSIL (1/7). All co-infections detected in HSIL cases involved HPV16 (75 %) (3/4) or HPV18 (25 %) (1/4). Only one case of triple infection with HPV16, HPV18 and HPV45 was detected in HSIL-positive samples.

**Table 2:** HPV prevalence and type distribution (*N* = 87^a^)

HPV type	n^b^ (%)	Single infection	Multiple infections
HPV-positive	52 (59.77)	30 (57.69)	22 (42.31)
High-risk (HR-HPV)			
HPV16	40 (76.92)	19 (47.5)	21 (52.5)
HPV18	11 (21.15)	4 (36.36)	7 (63.64)
HPV31	1 (1.78)	0	1 (100)
HPV33	13 (25.00)	2 (50)	9 (69.23)
HPV35	2 (3.57)	1 (50)	1 (50)
HPV45	7 (12.5)	0	7 (100)
HPV58	6 (10.71)	1 (16.67)	5 (83.33)
Low-risk (LR-HPV)			
HPV32	2 (3.85)	1 (50)	1 (50)
HPV62	1 (1.78)	0	1 (100)
HPV72	9 (17.31)	1 (11.10)	8 (88.90)
HPV81	1 (1.78)	0	1 (100)
HPV90	1 (1.78)	1 (100)	0
HPV87	1 (1.78)	0	1 (100)

^a^Number of tested cervical samples

^b^Number of subjects in a given category

Regardless of the cytological result, 50 % of HPV16-positive, 69.23 % of HPV18-positive and 86.6 7 % of HPV33-positive women were co-infected with at least one other HR- and/or LR-HPV type. HR-HPVs and LR-HPVs were in co-infection with more than one other HPV in 43.4 % and 88.5 % of cases, respectively. The detection rate of LR-HPV was 2.29 % with two main genotypes (HPV72 and HPV32). All other LR-HPV genotypes (32, 81, 87 and 90) were detected only once singly or in co-infection with other HPVs (Table 2). HPV90 was detected in single infection in LSIL samples, whereas the three other cases of LSIL were infected by HPV33, including one with a multi-infection by HPV16 and HPV58.

### Characterization of the E6/LCR sequence in HPV16

The E6 and LCR regions of the HPV16 genome were amplified and sequenced for 11 of the 22 detected HPV16 cases. Nucleotide (nt) changes were analyzed from nt 104 to 559 for the E6 gene and from nt 7384 to 7835 for the LCR sequence (Table 3). A comparison with the reference HPV16 sequence (GenBank accession number: K02718) showed that all sequences (E6 and LCR) contained at least one specific nucleotide variation. Fourteen nucleotide variations were found in the E6 region: nine non-synonymous mutations (G132C (R10T), G132T (R10I), C143G (Q14D), G145T (C51F), G255T (D64E), C256T (H78Y), T295G (L83V), C335T (H78Y) and T350G (L83V)) and five synonymous (silent) mutations (T109C, G196A, T286A, A289G and A403G). The non-synonymous mutations C143G, G145T, C335T and G132C/T that lead to Q14D and H78Y were the most frequent mutations (81.8 %) followed by the mutations R10T (54.5 %) and R10I (18.2 %), which are specific to African variants. Additional mutations (G196A, G255T, C256T, and T295G) inducing an amino-acid change (D64E) were all detected in only one sample. In addition, two samples showed a T-to-G mutation at position 350, specific to the European lineage.

**Table 3:** Nucleotide sequence variation in the E6 and LCR regions in HPV16 isolates

	E6 nucleotide position													LCR nucleotide position																	
Nucleotide position	109	132	143	145	196	255	256	286	289	295	335	350	403	7386	7393	7417	7435	7484	7489	7520	7668	7688	7713	7763	7785	7787	7825	7833	Variant	Class	Cytology
Predicted amino acid substitution	2 F	R10I/R10T	Q14D		V31	C51F		A61	V62	D64E	H78Y	L83V	L100																		
K02718	T	G	C	G	G	G	C	T	A	T	C	T	A	G	C	T	G	A	G	G	C	C	T	C	C	A	G	G			
CHUA130001	-	-	-	-		-	-	-	-	-	-	G																	E	G350	LSIL
CHUA130002														-	-	-	A	C	A	A	T	A	-	T	T	-	A	T	Af 2	-	ASC-US
CHUA 130003														-	-	-	A	C	A	A	T	A	-	T	T	-	A	T	Af 2	-	HSIL
CHUA 130012	-	-	-	-		-	-	-	-	-	-	G																	E	G350	carcinoma
CHUA 130026	-	C	G	T	-	-	-	a	g	-	T	-																	Af 1	1a	Normal
CHUA 130035	-	C	G	T	-	-	-	a	g	-	T	-	-	-	-	-	-	-	A	A	-	A	A	T	T	-	-	T	Af 1	1a	HSIL
CHUA 130021	c	T	G	T		-	-	a	g	-	T	-	g																Af 2	a	carcinoma
CHUA 130024	c	T	G	T	-	-	-	a	g	-	T	-	g																Af 2	a	ASC-US
CHUA 140002	c	T	G	T	-	-	-	a	g	-	T	-	g	C	-	-	-	C	A	A	T	A	-	T	T	-	A	T	Af 2	a	carcinoma
CHUA140010	c	T	G	T	-	-	-	a	g		T	-	g	C	-	-	A	C	A	A	T	A	-	T	T	-	A	T	Af 2	-	Normal
CHUL140009	c	T	G	T	-	-	-	a	g		T	-	g																Af 2	a	HSIL
CHUL140002	c	T	G	T	-	-	-	a	g		T	-	g	C	-	-	A	C	A	A	T	A	-	T	T	-	A	T	Af 2	a	HSIL
CHUL140016														-	T	-	-	-	A	A	-	A	A	T	T	-	-	T	Af 1	-	carcinoma
CHUA 130025	-	-	G	T	a	T	T	a	g	G	T	-	-	-	-	C	-	-	A	A	T	A	-	T	T	T	-	T	Af 1	-	carcinoma

(-): insufficient sequence to determine the class

Similarly, the LCR showed 15 nucleotide variations in eight samples. All eight samples showed the same mutation at six different sites: G7489A, G7520A, C7688A, C7763T, C7785T and G7833T. Six samples (75 %) shared the C7668T mutation, and five samples (62.5 %) contained the A7484C or G7825A mutations. The T7713A, C7417T and A7787T mutations were found in two samples each. All variation analyses based on the combination of the E6 gene and the LCR revealed that 85.7 % (12/14) and 14.7 % (2/14) belonged to the African and European branches, respectively. Af1 and Af2 were the most frequently identified variants with 33.3 % (3/9) and 55.6 % (5/9) in HSIL samples, respectively, and in equal proportion (40 % for each variant) in samples cytologically identified as carcinoma. Both European variants identified belonged to the G350 class.

## Discussion

This study reports the detection rate and the genotype-specific distribution of HPV among 87 women living in Libreville, Gabon presenting suspicious cervical abnormalities after a positive VIA/VILI test. These cervical abnormalities were confirmed by LBC in 40.23 % of cases, of which 40 % were HSIL. HPV DNA was detected in 59.77 % of the samples, with three main genotypes: HPV16, 18 and 33.

### Detection rate of HPV

Due to the difficulties in implementing cervical cytology-based screenings in developing or in low-income countries, VIA/VILI is particularly recommended for the early detection of cervical neoplasia [17]. Several studies have suggested that the specificity of VIA and Pap smear tests for early detection of CC are similar [5]. However, multiple parameters may affect VIA/VILI test accuracy from one health center to another, including lack of standardized protocols, differences in training of the health-care staff, lack of uniformity in application of gold standards for disease definition, and, finally, the absence of blind readings [6, 18]. Ultimately, inadequate conditions for one or several parameter(s) can give false-positive results. In our study only 40.23 % of positive VIA/VILI cases were confirmed by LBC. As noted in the literature, our data confirm the need to combine the VIA/VILI screening test with other histological/cytological and/or molecular assays such as HR-HPV testing. In fact, the predicted positive value of VIA is poor and needs triaging, such as HPV testing to improve the screening results — as observed in India [19] —, or histological screening as indicated by Mpiga et al., in Gabon [20].

The high detection rate of HPV (59.77 %) in this study among Gabonese women with cervical abnormalities was similar to those reported in previous studies conducted in Libreville [21] and in Bioko, Equatorial Guinea [22], which both found a prevalence of 60 %. However, another study conducted in Libreville among women attending an antenatal check-up or presenting general genital symptoms revealed a lower HPV prevalence rate (46 %) [11]. The difference in HPV prevalence between studies can be attributed to the study population and the laboratory methods used. Moreover, the HPV detection rate increases with the severity of cytological abnormalities: HPV was found in 100 % of HSIL samples. However, in most geographical regions, a peak of HPV prevalence is observed in women aged ≤ 25, followed by a decrease in older age groups. In our study, as expected in Africa and South America [23], an increased and stable HPV infection prevalence was observed among women aged 45 and older.

As observed in Equatorial Guinea and Gabon [21, 22], the most frequent HPV genotypes were HPV16, 18 and 33, in women presenting invasive cervical cancer, whereas HPV16, 18, 35, 56 and 82 were the genotypes most frequently found in Cameroon [24]. This difference may be partly attributable to the methods of HPV detection used. HPV detection methods vary in sensitivity according to the types of material, anatomical location, sets of primers used and the study population [25, 26]. In addition to the main HR-HPV genotypes detected, only one case of HPV90 was observed in LSIL. This has already been reported in a previous study on women in the USA [27]. Molecular investigation of this genotype based on the E6 gene showed a K16N mutation involved in p53 degradation that may explain its oncogenic potential in patients with negative Papanicolaou test [28].

### Analysis of the E6/LCR sequences in HPV16

The analysis of the nucleotide variation in the HPV sequence distinguished among HPV sub-types and can be used for epidemiological and transmission studies [29]. Villa et al. suggested that the distribution of HPV genotypes is associated with the ethnic group and geography [30]. Viral persistence and progression towards cervical cancer may be enhanced in HPV16 variants, which may partly explain the difference in prevalence in some populations [29]. Previous studies in Gabon and in other Sub-Saharan African countries have shown the predominance of the African lineage in HSIL cases [12, 31–34]. However, our analysis of the nucleotide variation of all HPV16 sequences showed that 85.7 % of the sequences belonged to the African branch (Af1 and Af2) and 14.3 % to the European branch. This European lineage in a HSIL+ case was identified for the first time in Gabon, whereas these non-African lineages have already been reported in northern and southern Africa. The introduction of this European lineage may be due to contacts between European and African populations. However, a larger female cohort is needed to test this hypothesis. All the mutations in the E6 and LCR sequences found in our study, such as the amino-acid substitutions Q14D, C51F, D64E and H78Y, which reduce intracellular binding to E6AP and degradation of the cellular tumor antigen p53, have already been described [16, 35–37]. Moreover, the nucleotide variations observed in the LCR sequence are known binding sites for various transcription factors, such as YY1, AP1, Oct-1 [38]. The G7520A mutation located at the YY1 binding site has been described in each cervical cancer patient worldwide [36] and is involved in the repression of HPV transcription and in quenching AP1 activity. We observed this nucleotide variation in all cases of carcinoma, but also in two women with normal cytology results.

A larger number of HPV16 sequences including other sequences or the complete genome would help to better identify the full panel of nucleotide variation and to determine their association with cervical lesions. Overall, our results are in agreement with the current data in the literature and confirm the importance of considering the African lineage. These molecular data of the E6 gene and the LCR region could be completed with those of the L1 gene. Indeed, Pande et al., showed that the A6695C mutation, which induces the replacement of threonine (polar uncharged) by proline (unpolar aliphatic), affected the structure or function of the L1 gene. This modification at the amino acid level may play an important role in immune recognition and vaccine development strategies [36].

### Limitations and strengths

The main limitation of this study was the small number of women included that limits the generalization of our data to the whole Gabonese and/or Central African populations. Furthermore, information on patient characteristics was not available for each participant, risk factors such as age at first intercourse or the number of sexual partners were not analyzed. Despite these limitations, this study shows the necessity to implement co-testing (VIA/L associated to HPV assay) in low-income countries, which could be considered an interesting alternative to VIA-cytology, currently used in Gabon.

## Conclusions

This study provides the first data on the HPV detection rate and molecular epidemiology among Gabonese women with a positive VIA/VILI test and cervical abnormalities confirmed by cytological analysis. HPV16, HPV18 and HPV33 were the most frequent genotypes found among these women. Furthermore, the analysis of HPV16 sequence variation in the E6 and LCR regions of the genome illustrate the predominance of the African variants in HSIL cases.

## Additional file

Additional file 1: Table S1. List of PCR primers sequences used. **Table S2.** Identified HPV genotypes per samples. (DOCX 287 kb)

## Abbreviations

ASC-US: Atypical squamous cells of undetermined significance
HIV: Human immunodeficiency virus
HPV: Human papillomavirus
HSIL: High-grade squamous intraepithelial lesions
LBC: Liquid-based cytology
LCR: Long control region
LSIL: Low-grade squamous intraepithelial lesions
VIA/VILI: Visual inspection with acetic acid and Lugol’s iodine

## References

[1] Bruni L. B-RL, Albero G., Aldea M., Serrano B., Valencia S., Brotons M., Mena M., Cosano R., Munoz J., Bosch FX., de Sanjosé S., Castellsagué X., ICO Information Centre on HPV and Cancer (HPV Information Centre). Human Papillomavirus and Related Diseases in the World. Summary report. 2014:12–8.

[2] El-Khatib Z, Tota JE, Kaufmann AM (2012). Progress on human papillomavirus (HPV) infection and cervical cancer prevention in sub-Saharan Africa: highlights of the 27th International Papillomavirus Conference in Berlin, 17-22 September 2011. J Epidemiol Global Health.

[3] Arbyn M, Castellsague X, de Sanjose S, Bruni L, Saraiya M, Bray F (2011). Worldwide burden of cervical cancer in 2008. Ann Oncol.

[4] Adefuye PO, Dada OA, Adefuye BO, Shorunmu TO, Akinyemi BO, Idowu-Ajiboye BO (2015). Feasibility, acceptability, and effectiveness of visual inspection of the cervix with acetic acid and cryotherapy for dysplasia in Nigeria. Int J Gynaecol Obstet.

[5] Urasa M, Darj E (2011). Knowledge of cervical cancer and screening practices of nurses at a regional hospital in Tanzania. Afr Health Sci.

[6] Vedantham H, Silver MI, Kalpana B, Rekha C, Karuna BP, Vidyadhari K (2010). Determinants of VIA (Visual Inspection of the Cervix After Acetic Acid Application) positivity in cervical cancer screening of women in a peri-urban area in Andhra Pradesh, India. Cancer Epidemiol. Biomark. Prev..

[7] Walboomers JM, Jacobs MV, Manos MM, Bosch FX, Kummer JA, Shah KV (1999). Human papillomavirus is a necessary cause of invasive cervical cancer worldwide. J Pathol.

[8] de Sanjose S, Quint WG, Alemany L, Geraets DT, Klaustermeier JE, Lloveras B (2010). Human papillomavirus genotype attribution in invasive cervical cancer: a retrospective cross-sectional worldwide study. Lancet Oncol.

[9] McDonald AC, Denny L, Wang C, Tsai WY, Wright TC, Kuhn L (2012). Distribution of high-risk human papillomavirus genotypes among HIV-negative women with and without cervical intraepithelial neoplasia in South Africa. PLoS One.

[10] Globocan. Cancer Fact sheets, 2012 Cervical Cancer. Available online: http://globocan.iarc.fr/Pages/fact_sheets_cancer.aspx. Accessed 11 Aug 2015.

[11] Si-Mohamed A, Ndjoyi-Mbiguino A, Cuschieri K, Onas IN, Colombet I, Ozouaki F (2005). High prevalence of high-risk oncogenic human papillomaviruses harboring atypical distribution in women of childbearing age living in Libreville, Gabon. J Med Virol.

[12] Assoumou SZB, L. M. A.; Mbiguino, A. N.; Mabika, B. M.; Belembaogo, E.; Khattabi, A.; Ennaji, M. M. Sequence variations of human papillomavirus type 16 E6 and E7 genes in cervical cancer isolates from Gabon. Br Microbiol Res J. 2015;8 (2):386-94. doi:10.9734/BMRJ/2015/17225.

[13] http://www.ncbi.nlm.nih.gov/books/NBK195239/ GWHOAf. WHO Guidelines for Screening and Treatment of Precancerous Lesions for Cervical Cancer Prevention. 2013. Accessed 11 Aug 2015.

[14] Sankaranarayanan R, Wesley R, Thara S, Dhakad N, Chandralekha B, Sebastian P (2003). Test characteristics of visual inspection with 4% acetic acid (VIA) and Lugol’s iodine (VILI) in cervical cancer screening in Kerala, India. Int J Cancer.

[15] Yamada T, Wheeler CM, Halpern AL, Stewart AC, Hildesheim A, Jenison SA (1995). Human papillomavirus type 16 variant lineages in United States populations characterized by nucleotide sequence analysis of the E6, L2, and L1 coding segments. J Virol.

[16] Huertas-Salgado A, Martin-Gamez DC, Moreno P, Murillo R, Bravo MM, Villa L (2011). E6 molecular variants of human papillomavirus (HPV) type 16: an updated and unified criterion for clustering and nomenclature. Virology.

[17] Organization WH (2013). WHO Guidelines for screening and Treatment of Precancerous Lesions for Cervical Cancer Prevention.

[18] Mahe C, Gaffikin L (2005). Screening test accuracy studies: how valid are our conclusions? Application to visual inspection methods for cervical screening. Cancer Causes Control.

[19] Basu P, Mittal S, Banerjee D, Singh P, Panda C, Dutta S (2015). Diagnostic accuracy of VIA and HPV detection as primary and sequential screening tests in a cervical cancer screening demonstration project in India. Int J Cancer.

[20] Mpiga E, Ivanga M, Koumakpayi IH, Engohan-Aloghe C, Ankely JC, Belembaogo E (2015). Interest in visual inspection with acetic acid and Lugol iodine with colposcope in screening of cervical lesions in Gabon. Pan Afr Med J.

[21] Zoa Assoumou S, Ndjoyi Mbiguino A, Mabika Mabika B, Nguizi Ogoula S, El Mzibri M, Khattabi A (2016). Human papillomavirus genotypes distribution among Gabonese women with normal cytology and cervical abnormalities. Infectious Agents Cancer.

[22] Garcia-Espinosa B, Nieto-Bona MP, Rueda S, Silva-Sanchez LF, Piernas-Morales MC, Carro-Campos P (2009). Genotype distribution of cervical human papillomavirus DNA in women with cervical lesions in Bioko. Equatorial Guinea Diagn Pathol.

[23] Smith JS, Melendy A, Rana RK, Pimenta JM (2008). Age-specific prevalence of infection with human papillomavirus in females: a global review. J Adolesc Health.

[24] Pirek D, Petignat P, Vassilakos P, Gourmaud J, Pache JC, Rubbia-Brandt L (2015). Human papillomavirus genotype distribution among Cameroonian women with invasive cervical cancer: a retrospective study. Sex Transm Infect.

[25] Qu W, Jiang G, Cruz Y, Chang CJ, Ho GY, Klein RS (1997). PCR detection of human papillomavirus: comparison between MY09/MY11 and GP5+/GP6+ primer systems. J Clin Microbiol.

[26] Gravitt PE, Peyton CL, Alessi TQ, Wheeler CM, Coutlee F, Hildesheim A (2000). Improved amplification of genital human papillomaviruses. J Clin Microbiol.

[27] Quiroga-Garza G, Zhou H, Mody DR, Schwartz MR, Ge Y (2013). Unexpected high prevalence of HPV 90 infection in an underserved population: is it really a low-risk genotype?. Arch Pathol Lab Med.

[28] Fu L, Van Doorslaer K, Chen Z, Ristriani T, Masson M, Trave G et al. Degradation of p53 by human Alphapapillomavirus E6 proteins shows a stronger correlation with phylogeny than oncogenicity. PloS One. 2010;5(9). doi:10.1371/journal.pone.0012816. 10.1371/journal.pone.0012816 PMC2941455 20862247

[29] Franco EL, Villa LL, Rahal P, Ruiz A (1994). Molecular variant analysis as an epidemiological tool to study persistence of cervical human papillomavirus infection. J Natl Cancer Inst.

[30] Villa LL, Sichero L, Rahal P, Caballero O, Ferenczy A, Rohan T (2000). Molecular variants of human papillomavirus types 16 and 18 preferentially associated with cervical neoplasia. J Gen Virol.

[31] Boumba LM, Qmichou Z, Mouallif M, Attaleb M, Mzibri ME, Hilali L (2015). Human papillomavirus genotypes distribution by cervical cytologic status among women attending the General Hospital of Loandjili, Pointe-Noire, Southwest Congo (Brazzaville). J Med Virol.

[32] Buonaguro FM, Tornesello ML, Salatiello I, Okong P, Buonaguro L, Beth-Giraldo E (2000). The uganda study on HPV variants and genital cancers. J Clinvirol.

[33] Qmichou Z, Khyatti M, Berraho M, Ennaji MM, Benbacer L, Nejjari C (2013). Analysis of mutations in the E6 oncogene of human papillomavirus 16 in cervical cancer isolates from Moroccan women. BMC Infect Dis.

[34] Qmichou ZMMEA, Mariam; El Mostafa El Fahime; Melloul, Marouane; Meriem Meftah El Khair; Mohammed El Mzibri; Meriem Khyatti; Mohammed Attaleb Molecular Characterization of HPV16 E6 and E7 Variants among Women with Cervical Cancer in Morocco. Br Microbiol Res J. 2013;3(4):692–705. doi:10.9734/BMRJ/2013/5465.

[35] Mosmann JP, Monetti MS, Frutos MC, Kiguen AX, Venezuela RF, Cuffini CG (2015). Mutation detection of E6 and LCR genes from HPV 16 associated with carcinogenesis. Asian Pac J Cancer Prev.

[36] Pande S, Jain N, Prusty BK, Bhambhani S, Gupta S, Sharma R (2008). Human papillomavirus type 16 variant analysis of E6, E7, and L1 genes and long control region in biopsy samples from cervical cancer patients in north India. J Clin Microbiol.

[37] Perez S, Cid A, Inarrea A, Pato M, Lamas MJ, Couso B (2014). Prevalence of HPV 16 and HPV 18 lineages in Galicia, Spain. PloS One.

[38] Bernard HU, Calleja-Macias IE, Dunn ST (2006). Genome variation of human papillomavirus types: phylogenetic and medical implications. Int. J. Cancer.

