# Supplementary material for: Molecular analysis of human Papillomavirus detected among women positive for cervical lesions by visual inspection with acetic acid/Lugol’s iodine (VIA/VILI) in Libreville, Gabon
Source: Infect Agent Cancer. 2016 Sep 7;11(1):50. doi: 10.1186/s13027-016-0098-1 (PMC5015258; doi:10.1186/s13027-016-0098-1)

# Supplementary data

Table S1. List of PCR primers sequences used.

| **Primers** | **Sequence (5’ to 3’)** |
| --- | --- |
| **GP5+** | TTT GTT ACT GTG GTA GAT ACT AC |
| **GP6+** | GAA AAA TAA ACT GTA AAT CAT ATT C |
| **MY09** | TCG-TCG-GCA-GCG-TCA-GAT-GTG-TAT-AAG-AGA-CAG-CGT-CCM-ARR-GGA-WAC-TGA-TC |
| **MY11** | GTC-TCG-TGG-GCT-CGG-AGA-TGT-GTA-TAA-GAG-ACA-GGC-MCA-GGG-WCA-TAA-YAA-TGG |
| **E-GP5+** | TCG-TCG-GCA-GCG-TCA-GAT-GTG-TAT-AAG-AGA-CAG-TTG-TTA-CTG-TGG-TAG-ATA-CTA-C |
| **E-GP6+** | GTC-TCG-TGG-GCT-CGG-AGA-TGT-GTA-TAA-GAG-ACA-GGA-AAA-ATA-AAC-TGT-AAA-TCA-TAT-TC |
| **HPVE6-F** | CGT-AAC-CGA-AAT-CGG-TTG-AAC |
| **HPVE6-R** | GCT-CAT-AAC-AGT-AGA-GAT-C |
| **HPVLCR-F** | CAA-CAC-CTA-CTA-ATT-GTG-TTG-TGG |
| **HPVLCR-R** | AAA-TCG-GTT-TGC-ACA-CAC-CCA-TGT |

**Table S2 Identified HPV genotypes per samples.** A genotype is identified regarding the minimal divergence among a set of read related to that particular genotype. Then other parameters such as the the divergence median and mean give an idea of the general distribution of the set of reads.  All parameters  assess  to which extent the taxonomic classification can be trusted.


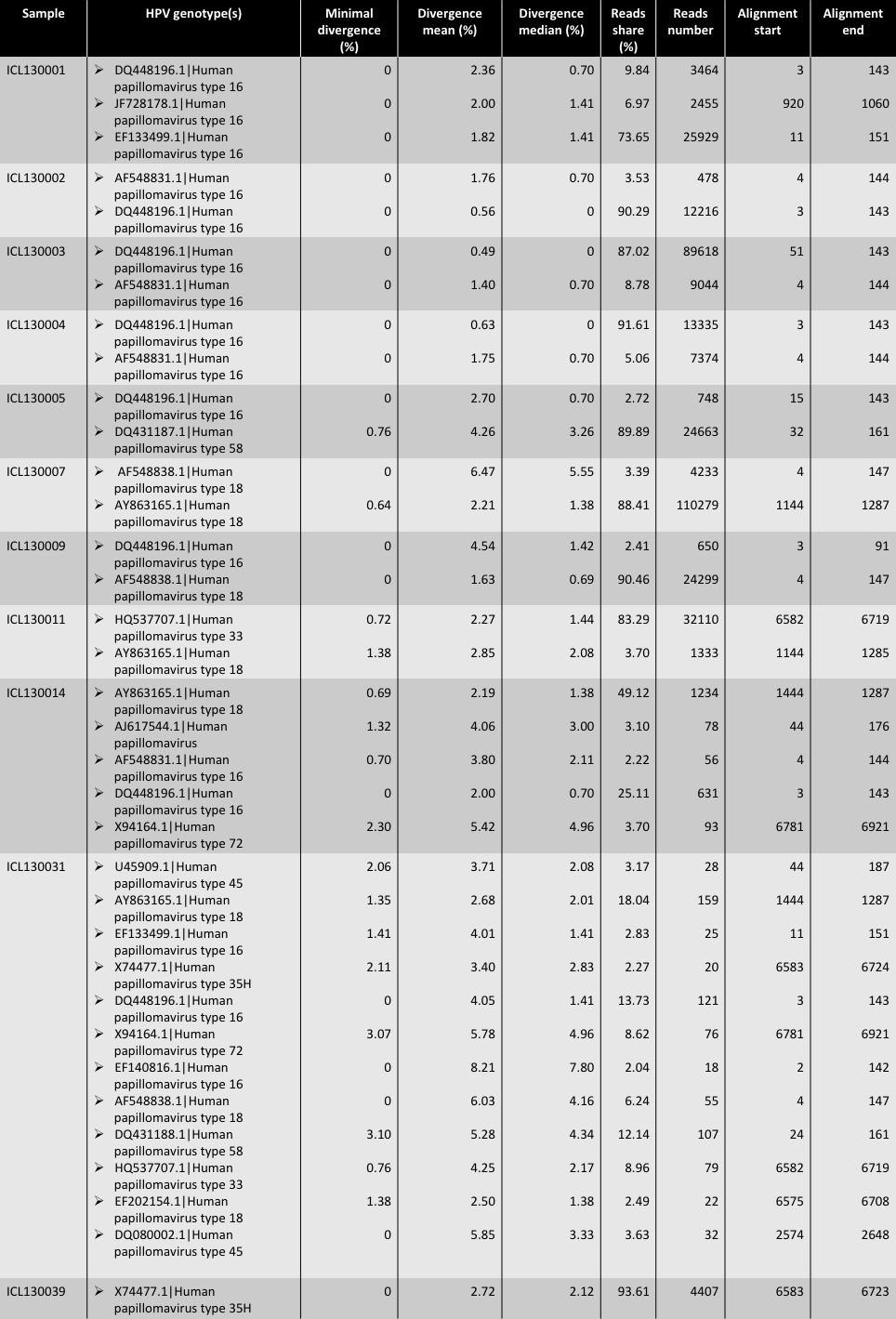

Supplement: Additional file 1: Table S1. — List of PCR primers sequences used. Table S2. Identified HPV genotypes per samples. (DOCX 287 kb) [file 13027_2016_98_MOESM1_ESM.docx]
